# A case of perforation of the prostatic abscess into the rectum resulting in a rectoprostatic fistula

**DOI:** 10.1002/iju5.12641

**Published:** 2023-09-19

**Authors:** Yuichi Sugiyama, Atsushi Fujikawa, Shuhei Yokokawa

**Affiliations:** ^1^ Department of Urology Yokosuka City Hospital Yokosuka Kanagawa Japan

**Keywords:** prostatic abscess, rectoprostatic fistula, transperineal catheter placement

## Abstract

**Introduction:**

A rectoprostatic fistula complicating a prostatic abscess is extremely rare, and there are many uncertainties regarding its treatment and prognosis.

**Case presentation:**

We present the case of a 68‐year‐old male who presented with rectal bleeding. Computed tomography reveals a prostatic abscess in contact with the rectum. As conservative treatment with antibiotics resulted in the aggravation of symptoms, we placed a transperineal catheter placement in the prostate, followed by drainage of the abscess through the catheter. The abscess communicated with the rectum, forming a rectoprostatic fistula. Subsequently, a transverse colostomy was performed. The abscess resolved, and no recurrence was observed even after 6 months of drainage. However, the rectal ulceration that resulted in the formation of the fistula persisted, and stoma closure was not achieved.

**Conclusion:**

In cases of prostatic abscess complicated by rectoprostatic fistula, drainage of the abscess along with colostomy seems essential for a curative approach.

**Abbreviations & Acronyms**
CT: computed tomography
DM: diabetes mellitus
MEPM: meropenem
MIC: minimum inhibitory concentration
PA: prostatic abscess
PSA: prostate‐specific antigen
RPF: rectoprostatic fistula

**Keynote message**

A 68‐year‐old male with DM had a PA that formed an RPF with the rectum, causing rectal bleeding. The patient was treated with antibiotics, catheter drainage, and colostomy. The PA resolved; however, the RPF persisted. Moreover, an RPF is rare and difficult to treat. Drainage and colostomy may be required to prevent infection recurrence.

## Introduction

An RPF complicated by PA is an extremely rare condition, with only a few reported cases, and its diagnosis and treatment are difficult to establish.^1^, ^2^, ^3^ However, even in cases of PA alone, the mortality rate is estimated to be approximately 1–16%, particularly in patients with risk factors such as DM leading to PA. Therefore, appropriate therapeutic intervention is necessary.^4^ We report a case in which drainage and colostomy was performed on a patient who developed severe PA resulting in RPF.

## Case report

**Fig. 1:**
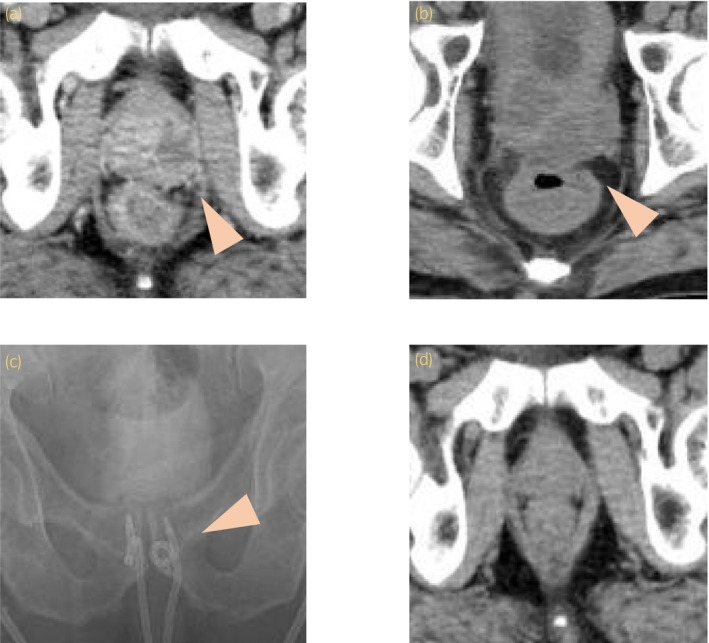
(a) PA (left lobe: 25 × 21 mm) was in contact with the rectum. (b) On CT during the exacerbation of inflammation, the abscess spread to both lobes of the prostate and was in contact with the rectal wall. (c) Two pigtail catheters were placed in the prostate. (d) CT of 6 months after the catheter removal shows improvement of the PA.

**Fig. 2:**
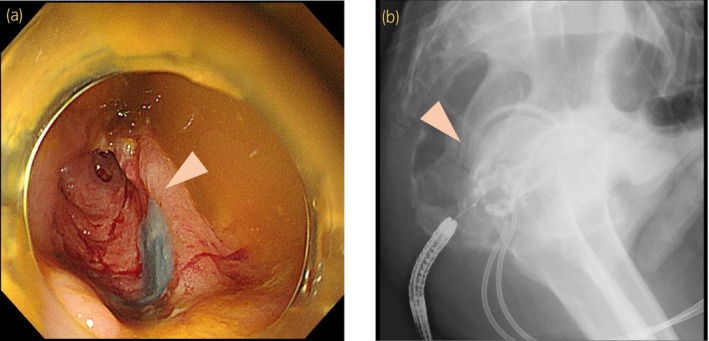
(a) Injection of indigo carmine the drain shows outflow from the margin of the rectal ulcer. (b) Injection of contrast agent from the outflow site of rectum confirmed the fistula between the prostate and rectum.

A 68‐year‐old male with a history of DM and hypertension presented for evaluation with a 3 week history of difficulty in defecation and a 1 week history of hematochezia. Blood tests revealed elevated inflammatory markers (WBC 8100/μL, CRP 16.58 mg/dL), and urine analysis revealed the presence of pyuria. As for DM, the patient had a fasting blood glucose level of 110 mg/dL and an HbA1c of 6.3%. Dietary therapy was performed, and no complications related to DM had developed. PSA levels were mildly elevated at 5.010 ng/mL. A urine culture revealed *Klebsiella pneumoniae*. CT scan suggested the formation of a 2 cm abscess in the left lobe of the prostate (Fig. 1a). Lower gastrointestinal endoscopy revealed an ulcer on the anterior wall of the lower rectum, without active bleeding. A biopsy of the rectal ulcer revealed infiltration of inflammatory cells into the rectal stroma, without any evidence of malignancy. Based on these findings, the patient was diagnosed with prostatitis and PA. Treatment was initiated with LVFX, as the identified organism *K. pneumoniae* exhibited a MIC of ≤0.12 for the drug. Rectal ulcer was observed, however, symptoms and inflammatory markers improved rapidly. However, on the 25th day, the patient developed a fever of 38°C and a rise in inflammatory markers (WBC 17 300/μL, CRP 23.28 mg/dL). Urine and blood cultures revealed *K. pneumoniae*. CT revealed prostate enlargement with an increased abscess size in the left lobe and the appearance of a new abscess in the right lobe (left lobe, 36 × 27 mm, right lobe, 24 × 13 mm). Furthermore, a part of the abscess was in contact with the rectal wall, suggesting possible adhesions or perforations (Fig. 1b). An echo‐guided perineal pigtail catheter was placed for diagnostic purposes and abscess drainage (Fig. 1c). Milky pus (approximately 30 mL) was drained from the major abscess cavities in both lobes. Urine and pus cultures revealed *K. pneumoniae*, consistent with previous findings, and showed resistance only to ABPC. Simultaneously, treatment with MEPM, which exhibited a MIC value of ≤0.25 against the causative pathogen, was initiated. To determine the presence of an a RPF fistula between the PA and the rectal ulcer, a repeat lower gastrointestinal endoscopy was performed. Indigo carmine was injected through the drain, and outflow from the margin of the rectal ulcer was observed (Fig. 2a). The contrast agent was injected from the outflow site, confirming the presence of RFP fistula between the prostate and rectum (Fig. 2b). The treatment with MEPM was continued, resulting in a prompt alleviation of inflammation. In the 2nd month, a transverse colostomy was performed to improve an RPF. The drainage from the catheter diminished significantly 5 days later, leading to its removal. Six months after catheter removal, CT revealed improvement in the PA and no signs of recurrent inflammation (Fig. 1d). No residual urine or any evidence of urinary dysfunction was observed. Urine analysis revealed normal results. The PSA level decreased to 1.005 ng/mL. However, the rectal ulcer that had formed RPF did not exhibit any improvement, and stoma closure was not achieved. It is anticipated that the stoma will need to be maintained indefinitely. Nevertheless, symptoms such as difficulty in defecation and hematochezia, which were present before the colostomy, had disappeared. No secretion or discharge was noted.

## Discussion

PA is a relatively rare complication of acute prostatitis. Ha *et al*. reported that DM as an underlying condition posed the highest risk for PA.^4^ It is estimated that more than 50% of the cases are associated with DM.^5^ The most common causative pathogen was *Escherichia coli* (27%), followed by *K. pneumoniae* (23%).^6^ Ludwig *et al*. reported treatment approaches for 18 self‐experimented cases of PA and it was discovered that 33% of patients exhibited limited improvement in symptoms with conservative therapy and required additional abscess drainage.^7^ Conservative management may be attempted for small and unilocular PAs; however, the choice of treatment should ultimately be determined based on individual conditions and circumstances. Furthermore, there are only a few reports on the formation of an RPF caused by a PA, indicating that it is rare. It has been reported as a complication of intravesical Bacillus Calmette‐Guérin therapy for bladder cancer.^1^ Additionally, RPF formation has been reported in xanthogranulomatous prostatitis.^2^ Regarding treatment for RPF, performing a colostomy followed by clipping of the rectal fistula was reported.^3^ However, based on available research, there are no accumulated case studies or established diagnostic and treatment approaches, suggesting that diagnosis and management should be considered inconclusive. In this case, during the initial gastrointestinal endoscopy, we were unable to detect any signs of a rectal ulcer forming a fistula with the PA, leading us to diagnose the PA and rectal ulcer as separate conditions. However, the fistula may have already formed at that time. We performed colostomy and prostatic drainage almost simultaneously, achieving favorable treatment outcomes in terms of PA recurrence inhibition. To the best of our knowledge, we could not find similar case reports. However, even after 6 months post‐drainage, the rectal ulcer persists, and closure of the stoma has not been achieved. Since rectal ulcer was already present at the initial colonoscopy, there remains controversial on whether more prompt drainage could have prevented stoma construction. Nevertheless, it is strongly suggested that regardless of the size of the abscess, catheter placement in the prostate or drainage through a perineal incision should be seriously considered.

## Conclusion

We described a case of a patient with DM as the underlying condition, in whom PA perforated and formed RPF. Abscess drainage and colostomy yielded favorable outcomes in terms of PA recurrence inhibition. However, considering the risk of inflammation exacerbation and RPF development, it appeared essential to always consider early drainage for PA.

## Author contributions

Yuichi Sugiyama: Writing – original draft; writing – review and editing. Atsushi Fujikawa: Supervision; writing – review and editing. Shuhei Yokokawa: Validation.

## Conflict of interest

The authors declare no conflict of interest.

## Approval of the research protocol by an Institutional Reviewer Board

Not applicable.

## Informed consent

The patient provided informed consent for publication of this case report.

## Registry and the Registration No. of the study/trial

Not applicable.

## References

[1] Eom JH , Yoon JH , Lee SW *et al*. Tuberculous prostatic abscess with prostatorectal fistula after intravesical bacillus Calmette‐Guérin immunotherapy. Clin. Endosc. 2016; 49: 488–491. 26978160 10.5946/ce.2015.145 PMC5066407

[2] Xing L , Liu Z , Deng G *et al*. Xanthogranulomatous prostatitis with prostato‐rectal fistula: a case report and review of the literature. Res. Rep. Urol. 2016; 8: 165–168. 27695691 10.2147/RRU.S110658 PMC5033499

[3] Ubrig B , Schmidt‐Heikenfeld E , Degener S , Roosen A , Boy A . Minimally invasive repair of a prostatorectal fistula with an over‐the‐scope rectal clip. J. Endourol. Case Rep. 2017; 3: 146–148. 29098198 10.1089/cren.2017.0097 PMC5665494

[4] Ha US , Kim ME , Kim CS *et al*. Acute bacterial prostatitis in Korea: clinical outcome, including symptoms, management, microbiology and course of disease. Int. J. Antimicrob. Agents 2008; 31: S96–S101. 18065208 10.1016/j.ijantimicag.2007.07.041

[5] Ackerman AL , Parameshwar PS , Anger JT . Diagnosis and treatment of patients with prostatic abscess in the post‐antibiotic era. Int. J. Urol. 2018; 25: 103–110. 28944509 10.1111/iju.13451

[6] Lee DS , Choe HS , Kim HY *et al*. Acute bacterial prostatitis and abscess formation. BMC Urol. 2016; 16: 38. 27388006 10.1186/s12894-016-0153-7 PMC4936164

[7] Ludwig M , Schroeder‐Printzen I , H G Schiefer HG , Schiefer HG , Weidner W . Diagnosis and therapeutic management of 18 patients with prostatic abscess. Urology 1999; 53: 340–345. 9933051 10.1016/s0090-4295(98)00503-2

